# Ecology of Nitrogen Fixing, Nitrifying, and Denitrifying Microorganisms in Tropical Forest Soils

**DOI:** 10.3389/fmicb.2016.01045

**Published:** 2016-07-05

**Authors:** Silvia Pajares, Brendan J. M. Bohannan

**Affiliations:** ^1^Unidad Académica de Ecología y Biodiversidad Acuática, Instituto de Ciencias del Mar y Limnología, Universidad Nacional Autónoma de MéxicoCoyoacán, Mexico; ^2^Institute of Ecology and Evolution, University of Oregon, EugeneOR, USA

**Keywords:** nitrogen processes, tropical forest soils, soil microbial community, nitrogen functional genes, nitrous oxide

## Abstract

Soil microorganisms play important roles in nitrogen cycling within forest ecosystems. Current research has revealed that a wider variety of microorganisms, with unexpected diversity in their functions and phylogenies, are involved in the nitrogen cycle than previously thought, including nitrogen-fixing bacteria, ammonia-oxidizing bacteria and archaea, heterotrophic nitrifying microorganisms, and anammox bacteria, as well as denitrifying bacteria, archaea, and fungi. However, the vast majority of this research has been focused in temperate regions, and relatively little is known regarding the ecology of nitrogen-cycling microorganisms within tropical and subtropical ecosystems. Tropical forests are characterized by relatively high precipitation, low annual temperature fluctuation, high heterogeneity in plant diversity, large amounts of plant litter, and unique soil chemistry. For these reasons, regulation of the nitrogen cycle in tropical forests may be very different from that of temperate ecosystems. This is of great importance because of growing concerns regarding the effect of land use change and chronic-elevated nitrogen deposition on nitrogen-cycling processes in tropical forests. In the context of global change, it is crucial to understand how environmental factors and land use changes in tropical ecosystems influence the composition, abundance and activity of key players in the nitrogen cycle. In this review, we synthesize the limited currently available information regarding the microbial communities involved in nitrogen fixation, nitrification and denitrification, to provide deeper insight into the mechanisms regulating nitrogen cycling in tropical forest ecosystems. We also highlight the large gaps in our understanding of microbially mediated nitrogen processes in tropical forest soils and identify important areas for future research.

## Introduction

Nitrogen (N) is an important component of proteins and nucleic acids, and thus is an essential nutrient for all organisms. Furthermore, the N supply to organisms regulates primary productivity in many natural ecosystems. N exists in multiple oxidation states and chemical forms in nature, and transformations among these different forms are typically catalyzed by microorganisms (Hayatsu et al., 2008). Soil microorganisms constitute a major portion of the biodiversity in soils and play a pivotal role in soil processes (Fierer and Jackson, 2006; Schimel and Schaeffer, 2012; Philippot et al., 2013), which ultimately affect the functioning of terrestrial ecosystems. Given the biogeochemical contributions of microbes, microbial community dynamics may be associated with temporal and spatial variation in N processes and rates. Thus, understanding the link between biogeochemical N processes and microbial community dynamics can provide a more mechanistic understanding of the N cycle than the direct observation of N dynamics. Likewise, it is important to know the factors that influence the biodiversity and functioning of soil microbes that mediate these processes in order to predict ecosystem responses to a changing environment (Singh et al., 2010).

Tropical forests are particularly important in the N cycle. Although these ecosystems occupy only 12% of the Earth’s surface, they contribute ∼70% of terrestrial N fixation and emit ∼50% of nitrous oxide (N_2_O; Townsend et al., 2011). They are a major reservoir of biodiversity and play a vital role in regulating the Earth’s climate and biogeochemical cycles through their vast exchanges of energy, water, and nutrients with the atmosphere (Foley et al., 2003; Bonan, 2008). Tropical forests account for nearly 40% of terrestrial net primary production, contain 25% of the world’s biomass carbon (C) and play a critical role in buffering the atmosphere against rising carbon dioxide (CO_2_) (Townsend et al., 2011; Wright, 2013). On the other hand, these forest ecosystems are threatened by high levels of deforestation, rapid rates of land conversion to agriculture, and chronic-elevated N deposition, with important implications for global climate (Stork et al., 2009; Hietz et al., 2011). As a result, there have been increased efforts recently to understand and predict how soil microbial communities in tropical forests regulate biogeochemical processes, in particular N cycling.

Our understanding of biological N cycling in forest ecosystems has undergone a major shift in the past decade in concert with the growing interest in integrating evolutionary and ecological theory into microbial ecology (Prosser et al., 2007; Fierer et al., 2009b; Philippot et al., 2010), the rapid development of molecular-based culture-independent methods (Roesch et al., 2007; Caporaso et al., 2011; Palmer et al., 2012), and the use of N isotope tracer techniques (Baggs, 2008; Kool et al., 2010). These advances have increased our understanding of microbial communities, their functional potential, and their physiological state (Houlton et al., 2006; Roesch et al., 2007; Levy-Booth et al., 2014), and have sparked a new interest in the functional link between soil microbial community structure and N cycling (Cusack et al., 2011; Treseder et al., 2011; Philippot et al., 2013). As a result, we are beginning to understand the microbial community ecology involved in N processes in forest ecosystems, which provide us with a more complete picture of the regulation of the N cycle and its response to environmental change (Bottomley et al., 2012; Levy-Booth et al., 2014; Zhang et al., 2014).

Although some excellent reviews of the ecology of N-cycling microorganisms in soils exist (e.g., Wallenstein et al., 2006; Philippot et al., 2007; Hayatsu et al., 2008; Braker and Conrad, 2011; Levy-Booth et al., 2014), these focus primarily on temperate systems, and the relationship between N processes and microbial community dynamics in tropical forest soils remains poorly understudied. In this review we describe and summarize currently available information regarding the relationship between soil microbial ecology and three relevant N-cycling processes that govern the fate of N in tropical forest soils. Although we also briefly introduce other important microbial N processes, we focus on the N-fixer (diazotrophs), nitrifier and denitrifier communities because these functional groups have been the most studied in tropical soils. We close our review with a discussion of the impact of anthropogenic environmental changes on the microbially mediated mechanisms regulating N cycling in tropical forest ecosystems.

## The Nitrogen Cycle in Tropical Forest Soils

Tropical soils are generally on older geologic substrates depleted of rock-derived elements such as phosphorus (P) and cations. As a consequence, these soils are typically acidic (pH < 5) and their fertility is often dependent on the cycling of a thin layer of organic matter associated with the large amount of plant litter material (Hall and Matson, 2003; Macrae et al., 2013). Thus, in contrast to temperate forests, tropical forests on highly weathered soils are assumed to be primarily limited by P and “N-saturated” (Hedin et al., 2009; Cleveland et al., 2011), leading to an open dynamic N cycle, with relatively high levels of N loss via denitrification and leaching (Vitousek and Matson, 1988; Martinelli et al., 1999; Brookshire et al., 2012) balanced by high levels of N fixation (Reed et al., 2007; Cusack et al., 2009). This assumption is also supported by observations of high plant and soil N/P ratios, high abundance and diversity of legume plants, and the export of bioavailable N at the ecosystem scale (Supplemenatary Table S1; Houlton et al., 2006; Hedin et al., 2009; Vitousek et al., 2010). For example, some fertilization experiments conducted in tropical forests have demonstrated a stronger response of plant biomass to added P than to added N, supporting the paradigm that tropical ecosystems on weathered soils are predominantly P limited (Tanner et al., 1998; Harrington et al., 2001). However, there is emerging evidence of limitation and colimitation by other nutrients, such as N and potassium, in tropical forests (Kaspari et al., 2008; Wullaert et al., 2010; Wright et al., 2011). For instance, strong colimitation by N and P of vegetation and soil organisms has been observed in an Ecuadorian montane forest (Wullaert et al., 2010; Homeier et al., 2012). In this forest ecosystem, moderate additions of N (50 kg ha^-1^ yr^-1^) and P (10 kg ha^-1^ yr^-1^) led to not only an increase in foliar N and P concentrations, but also altered soil microbial biomass, standing fine root biomass, stem growth, and litterfall (Homeier et al., 2012). Additionally, forest disturbance may lead to N limitation of young secondary forests (Davidson et al., 2007). Research to date suggests that nutrient limitation in these ecosystems is likely not a question of N vs. P, but instead is the result of complex interactions among multiple nutrient cycles and their linkages with biological processes (Townsend et al., 2011; Alvarez-Clare et al., 2013).

Soil N transformations in tropical forests are affected by unique soil characteristics (e.g., low pH, rapidly fluctuating redox conditions and large amounts of Fe oxides, plant litter material and available N content) and environmental conditions (e.g., high humidity and low annual fluctuation in temperature), making it difficult to identify the main pathways and mechanisms affecting process-specific transformations (**Supplementary Table S1**; Vitousek and Matson, 1988; Hedin et al., 2009; Xu et al., 2013; Gao et al., 2015). For example, resource stoichiometry influences microbial growth rates (Rousk and Bååth, 2007) and the production of extracellular enzymes that degrade soil organic matter (Mooshammer et al., 2012). However, it remains unclear if the pattern of nutrient limitation documented for plants (i.e., strong limitation by P) in tropical forests holds true for soil microbes. For instance, there is evidence that P can limit microbial growth (Turner and Joseph Wright, 2014) and mineralization (Cleveland and Townsend, 2006; Kaspari et al., 2008) in lowland tropical forests found on heavily weathered soils. In contrast, soil microbial biomass and N mineralization in tropical montane forests on younger soils seem to be constrained primarily by N limitation (Hall and Matson, 2003; Cusack et al., 2010). Furthermore, it has been shown that the response of soil microorganisms to nutrient addition in an Ecuadorian montane rainforest varies with both altitude and duration of nutrient addition (Krashevska et al., 2014). Thus, nutrient availability to soil tropical communities can be affected by multiple factors such as elevation, temperature, rainfall and differences in parent material.

**FIGURE 1:**
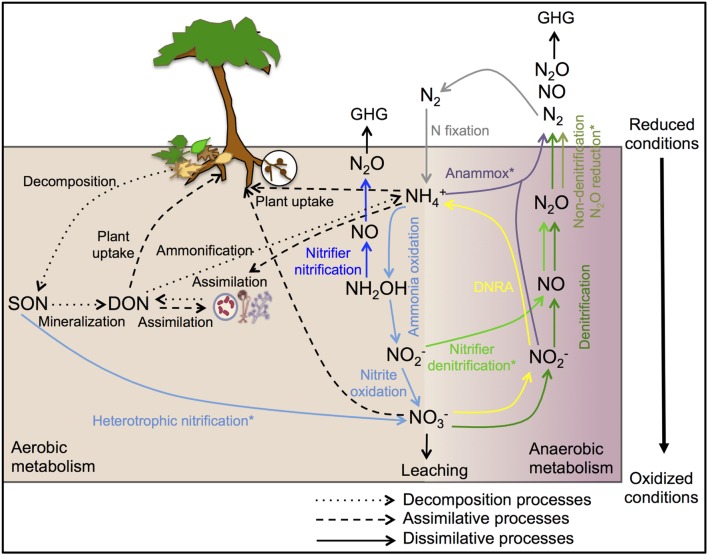
**Schematic representation of the biological N cycle in tropical forest soils.** The arrows of decomposition, assimilative and abiotic processes are shown in black and the arrows of dissimilative processes are shown in different colors: N-fixation (gray), nitrification (light blue), nitrifier nitrification (dark blue), nitrifier denitrification (light green), denitrification (dark green), non-denitrification N_2_O reduction (olive green), DNRA (yellow), and anammox (purple). DNRA, dissimilatory nitrate reduction to ammonium; SON, soil organic N; DON, dissolved organic N; GHG, greenhouse gas. ^∗^Occurrence and ecology of these processes in tropical forest soils has not been yet investigated.

The complexity of the biological N cycle in tropical forest soils could be depicted by a simplified model (**Figure 1**) that consists of three main processes (Levy-Booth et al., 2014): (1) Decomposition of plant litter and dead organisms to soil organic matter, which can be further degraded to dissolved organic N and ammonium (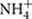); (2) assimilative processes of dissolved organic N, nitrate (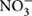) and 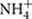 by plants and microorganisms for growth and replication; and (3) dissimilative processes including N fixation, nitrification, denitrification, and dissimilatory nitrate reduction to ammonium (DNRA), as well as newly described pathways such as codenitrification and anammox. The pool sizes and rates of production of N available to soil microbes and plants are much greater in many tropical forest soils than in temperate counterparts, due to high rates of N fixation by free-living microbes and rhizobia associated with legumes, which are especially abundant in many tropical ecosystems (Hedin et al., 2009). This high N availability can exceed the ecosystem N retention capacity and induce high rates of dissimilative processes, with N losses by 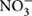 leaching and emissions of nitric oxide (NO) and N_2_O (Hall and Matson, 1999; Hedin et al., 2003; Biggs et al., 2004; Brookshire et al., 2012).

The N_2_O released from soil is of particular concern because it is a potent greenhouse gas. Tropical forest soils are considered the largest natural source of N_2_O with a source strength of 3.0 Tg N yr^-1^ (Werner et al., 2007a). The production of N_2_O in soil has traditionally been assumed to result from the processes of nitrification and denitrification, but growing evidence suggests that other processes may be involved, including nitrifier denitrification (Wrage et al., 2001), non-denitrifying N_2_O reduction (Sanford et al., 2012), DNRA (Tiedje, 1988), anaerobic ammonium oxidation (anammox; Mulder et al., 1995), and co-denitrification (Spott et al., 2011). Understanding the factors that determine the relative contributions of these different processes (and the microbes that mediate them) to N_2_O flux has important implications for climate change. However, it is still not clear what underlying processes drive gaseous N production from tropical forest soils. For instance, it has been suggested that nitrification is an important source of N_2_O in Amazon forest soils (Neill et al., 2005). However, some studies conducted in tropical forest ecosystems in Australia (Butterbach-Bahl et al., 2004), Kenya (Werner et al., 2007b), and Rwanda (Gharahi Ghehi et al., 2012) suggest that denitrification and nitrifier denitrification could be the main pathways of N_2_O gas production rather than nitrification. Likewise, anammox bacteria have been detected in a wide range of soil environments (Humbert et al., 2010; Sonthiphand et al., 2014), but the role of these organisms in N loss from tropical forest soils has yet to be determined. The DNRA process has also been suggested as a possibly important pathway for 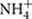 production in these soils (Silver et al., 2001), because the occurrence of DNRA is generally favored by low and fluctuating redox conditions, such as those in humid tropical forest soils (Pett-Ridge et al., 2006; Templer et al., 2008). For instance, Templer et al. (2008) found that the rate of DNRA (∼35% of the gross nitrification rate) in a humid tropical forest soil in Puerto Rico was much higher than the rate of N_2_O production from denitrification.

## N-Cycling Microbes in Tropical Forest Soils

There is evidence that tropical forest soils may harbor novel microbial communities, such as unique clades of Acidobacteria (Kim et al., 2007) and Verrucomicrobia (Ranjan et al., 2015). However, these soils are an especially understudied microbial environment, and very few studies have explored the immense diversity of soil microorganisms in tropical forests (Kim et al., 2007; Bruce et al., 2010; Ranjan et al., 2015) or asked if they are phylogenetically or physiologically distinct from those in temperate soils (Fierer and Jackson, 2006; Fierer et al., 2012b; Ramirez et al., 2012). The majority of these studies have concentrated on the effects of land-use conversion on the structure and function of soil microbial communities (e.g., da Jesus et al., 2009; Lee-Cruz et al., 2013; Rodrigues et al., 2013; Paula et al., 2014; Ranjan et al., 2015). Few studies have examined seasonal influences of precipitation (Krashevska et al., 2012; McGuire et al., 2012), or the effects of plant species diversity (Carney and Matson, 2006; McGuire et al., 2012) or N addition (Cusack et al., 2010, 2011; Isobe et al., 2012; Liu et al., 2013a) on tropical soil microbial communities.

Linking microbial community composition, abundance, and activity to rates and controls of biogeochemical transformations is challenging in any ecosystem, but is especially challenging in tropical forest soils. This is due not only to the complexity of tropical forest microbial communities, but also to the high degree of heterogeneity in plant functional diversity and soil chemistry often found in tropical forest ecosystems. For example, rates of many microbially mediated N processes show marked substantial variation across small temporal and spatial scales in tropical forest soils (Silver et al., 2001; Davidson et al., 2004; Pett-Ridge et al., 2006). Tropical plant community composition varies strongly across space in many tropical forest ecosystems, and this can contribute to the high degree of heterogeneity in N cycle processes observed in these systems. For instance, the large amount of plant litter and rhizodeposition (the release of C compounds from living plant roots) in tropical forest soils may enhance microbial activity and stimulate production of microbial exoenzymes that “mine” for more complex soil organic N compounds (Paterson, 2003). The N cycle is also particularly susceptible to changes in soil redox conditions, which are highly variable in tropical forest soils and can cycle between oxic and anoxic on a scale of hours to days (Silver et al., 2001). These fluctuating soil redox regimes are a critical determinant of microbial metabolism (DeAngelis et al., 2010), and may facilitate the co-occurrence of microbial N transformations with significantly different sensitivities to soil O_2_ availability, such as nitrification (oxic), denitrification (hypoxic), and DNRA (anoxic). Pett-Ridge et al. (2006) found that the rapidly fluctuating redox conditions in a humid tropical forest soil in Puerto Rico allow anoxic and oxic N processes to co-occur, which are mediated by tolerant and resilient microbial communities adapted to nutrient resources that are spatially and temporally variable.

A comprehensive understanding of the microbially mediated tropical N cycle is critical because it affects our knowledge of how tropical forests function within the integrated Earth-climate system. For example, quantification and characterization of microbial functional genes involved in N cycling (**Table 1** and **Supplementary Table S2**) can help create informative models of N-cycling rates and their feedback with C dynamics, as well as the impact of N_2_O emissions from these soils, providing predictions of, and mitigation strategies for, greenhouse gas emissions (Levy-Booth et al., 2014; Lammel et al., 2015). We discuss below currently available information regarding microbial functional groups that control the most commonly studied N processes (N fixation, nitrification, and denitrification), with an emphasis on unique aspects of their ecology in tropical forest soils.

**Table 1:** Studies using N functional genes related to N fixation, nitrification, and denitrification processes in tropical forest soils.

N genes	Methods	Ecosystem	Edaphic characteristics	Condition tested	Major relationships in N functional genes	Reference
*nifH, amoA, nirK, nirS, norB, nosZ*	*In situ* measure of GHG, qPCR	PTF, pasture and crops in Amazonia (Brazil)	Clay Oxisol, pH 3.9–5.1, 0.23–0.29% TC, 0.01–0.02% TN	Influence of land use change on microbial gene abundance and GHG emissions	*nifH* and AOB are more abundant in forest soils. AOA dominates in all sites. *amoA* genes correlate with 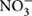 . PTF soils have the highest abundances of denitrifiers, which correlate with increased N_2_O emissions	Lammel et al., 2015
*nifH, amoA, nirS, nosZ*	DGGE, qPCR	Humid TFs (Puerto Rico, USA)	Oxisol and Inceptisol, pH 4.1–6.3, 0.01–0.2% TC, 0.001–0.06% TN	Influence of parent material, forest types and soil depth on bacterial community structure and N functional genes	N genes decline significantly with soil depth. *nifH, nirS*, and *nosZ* are more abundant in the Inceptisol soil. *nirS* and *nosZ* have the highest abundance and AOB has the lowest abundance	Stone et al., 2015
*amoA*	^15^N isotope technique (N transformations), qPCR	Evergreen subtropical forests (China)	Sandy loam Ferralsols and Cambisols, pH 4.2–5.8, 1.43–2.09% TC, 0.08–0.1% TN	Effect of Fe oxide and organic substrate addition on soil N transformations and *amoA* genes	Decrease of *amoA* abundance and GN and increase of 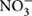 immobilization is caused by high Fe oxide content rather than low pH	Jiang et al., 2015
*nifH, amoA, hao, narG, nirK, nirS, norB, nosZ*	GeoChip Microarray	Rain PTFs (China)	pH 4.4, 4.7–5.6% TC, 0.18–0.23% TN	Influence of environmental factors and chronic N deposition on functional gene diversity	High relative abundance of *amoA* and *narG* due to the high soil available N, which could be caused by the high rate of N deposition	Cong et al., 2015
*nifH, amoA, hao, narG, nirK, nirS, norB, nosZ*	GeoChip Microarray	PTF, STF and pasture in Amazonia (Brazil)	Sandy loam podzolic Latosol, pH 4.0–4.7, 1.43–2.02% TC, 0.1–0.15% TN	Influence of land use change on functional gene diversity, composition, and abundance	Nitrification genes are more abundant in forest sites than in pasture. *nifH, narG, nirS*, and *norB* were linked to both forest sites. Genes changes correlate with pH, Fe availability, texture, and organic matter	Paula et al., 2014
*nifH*	Clone libraries, qPCR	PTF, STF and pasture in Amazonia (Brazil)	Sandy loam podzolic Latosol, pH 4.6–5.8, 0.07–0.08% TC, 0.006–0.02% TN	Influence of land use change on free-living N-fixing microorganisms	*nifH* composition changes and its abundance increases following forest-to-pasture conversion due to changes in soil pH, TN, and C/N	Mirza et al., 2014
*narG, nirK, norB, nosZ*	Acetylene inhibition method (PD), qPCR	Conifer subtropical STF, shrub forest and farmland (China)	Acrisols and Ferralsols, pH 4.4–5.3, 0.95–2.47% TC, 0.08–0.22% TN	Influence of land use on denitrifiers abundance and total N gas production	PD, N gas production, and denitrifying genes were affected by land use change. PD, *norB*, and *nosZ* were positively correlated with pH	Yu et al., 2014
*narG, nirK, norB, nosZ*	Acetylene inhibition method (PD), qPCR, clone libraries	Temperate and subtropical forests (China)	Temperate: pH 4.5–7.4, Eh 650 mV; subtropical: pH 4.3–6.8, Eh 600 mV	The mechanisms governing low denitrification capacity and high N_2_O emissions in subtropical forests soils	High Eh induced low denitrification capacity in subtropical soils (activity reduction of *nosZ*), being NO and N_2_O the dominant gas products	Zhang et al., 2014
*amoA*	Shaken slurry method (PN), TRF profiles, clones libraries, qPCR	Seasonal evergreen TF and grassland (Trinidad)	Soils from different parent materials, pH 4.8–8.2, 0.39–2.98% TC, 0.06–0.35% TN	Influence of edaphic drivers on PN and nitrifying community structure	Soil N characteristics are significant for AOA, but not for AOB, and pH is not a major drive for AOA and AOB	de Gannes et al., 2014
*amoA*	Shaken slurry method (PN), clones libraries, qPCR	Humid TF (Puerto Rico, USA)	High weathered clay loam Ultisols, pH 3.9–5.4	Influence of oxic/anoxic fluctuation on PN and ammonia oxidizers	AOA community is tolerant to extended periods of anoxia. AOB were not detected	Pett-Ridge et al., 2013
*narG, nirK, nirS, nosZ*	*In situ* measure of N_2_O, acetylene inhibition method (PD), qPCR	Native wet sclerophyll forest (Australia)	Sandy Alfisol, pH 4.6–5.5, 3.5–6.9% TC; 0.10–0.33% TN	Effect of long-term repeated burning on N_2_O flux, key soil properties, and denitrification gene abundance	More frequent fire reduced N_2_O fluxes and C and N availability. Fire treatments did not significantly affect denitrification genes abundance	Liu et al., 2013b
*amoA*	^15^N isotope technique (GN), clone libraries, culture, qPCR	Humid subtropical forests (China)	Sandstone Oxisol, pH 3.8–4.0, 2.6–4.6% TC, 0.1–0.19% TN	Influence of chronic N deposition on activity and composition of nitrifying community	Extraordinary abundance of AOA. AOB were not detected. Significant correlation between AOA abundance and GN rates.	Isobe et al., 2012
*amoA*	Shaken slurry method (PN), T-RFLP, clones libraries, qPCR	Tea orchard soils and pine subtropical forest (China)	Ultisol, pH 3.6–6.3, 0.003–0.071% TN	Long-term effects of low pH and N fertilization on the abundance, composition, and activity of AOA and AOB	AOA dominates in all sites and nitrification is driven by AOA in these acidic soils.	Yao et al., 2011
					Specific AOA and AOB populations occupy distinct pH niches.	
*nifH*	Acetylene inhibition method (NFR), clone libraries, qPCR	Lowland rain TF (Costa Rica)	P-poor Ultisols	Links between N-fixer community structure from leaf litter, and changing P availability	P addition increased N fixation rates, N-fixers diversity and relative abundance, and the efficiency of N-fixers	Reed et al., 2010
*amoA*	Shaken slurry method (PN), clone libraries, PLFA	Atlantic lowland TF (Costa Rica)	Sandy loam soils, pH 5.2–5.8, 3.1–4.6% TC	Land-use types and plant diversity influence on AOB community	AOB differs among land-use types, but not across plant diversity, and correlates with PN	Carney et al., 2004


*TF, tropical forest; PTF, Primary tropical forest; STF, Secondary tropical forest; NFR, N fixation rate; PN, potential nitrification; GN, gross nitrification; PD, potential denitrification; TC, total carbon; TN, total nitrogen; Eh, redox potential; DGGE, denaturant gradient gel electrophoresis; TRF, terminal restriction fragment; T-RFLP, terminal restriction fragment length polymorphism; qPCR, real-time PCR; AOA, ammonia- oxidizing archaea; AOB, ammonia- oxidizing bacteria; PLFA, phospholipid fatty acid composition, GHG, greenhouse gas.*

### Microbes Involved in N Fixation

Biological N fixation is the largest natural source of new N to most terrestrial ecosystems (Galloway et al., 2004). In this process symbiotic and free-living diazotrophic microorganisms reduce atmospheric dinitrogen gas (N_2_) to reactive and biologically available forms. The *nifH* gene, encoding the reductase subunit of nitrogenase, the enzyme that catalyzes this reaction, has been widely used as a genetic marker to study the diversity and abundance of diazotrophs (Zehr et al., 2003; Gaby and Buckley, 2011). Likewise, a wide variety of PCR primer sets from multiple diazotrophic bacteria have been used to characterize and quantify the *nifH* gene in soils (Gaby and Buckley, 2012).

Relationships between diazotroph communities and N fixation rates have been investigated in many terrestrial ecosystems, including temperate forest soils (Rosch et al., 2002; Yeager et al., 2005), wetlands (Moseman et al., 2009), desert systems (Yeager et al., 2004; Wang et al., 2016), grasslands (Tu et al., 2016), and agricultural lands (Pereira e Silva et al., 2013). Despite the fact that tropical forest ecosystems maintain considerable biological N fixation to balance the large potential N losses, this process and the microbial community involved in it remain poorly investigated in these soils (Cusack et al., 2009; Reed et al., 2010; Mirza et al., 2014). Studies of lowland tropical rainforest in Costa Rica reported that soils were dominated by *nifH* clones related to the genera *Heliobacterium* (a member of the Firmicutes), as well as *Gluconacetobacter*, *Methylobacterium*, *Azospirillum*, and *Zymomonas* (all Alpha-proteobacteria; Reed et al., 2010). Diazotroph communities in primary and secondary rainforest soils in the western Amazon Basin of Brazil have been reported to be mainly composed of ubiquitous and abundant members of the Alpha and Beta-proteobacteria (e.g., the genera *Azospirillum*, *Azorhizobium*, *Bradyrhizobium*, *Methylobacterium*, *Burkholderia*), Firmicutes (e.g., *Paenibacillus*, *Heliobacterium*), and Cyanobacteria (e.g., *Nostoc*, *Anabaena*; Mirza et al., 2014). Interestingly, *nifH* sequences associated with methanogenic Archaea (*Methanoregula*, *Methanosphaerula*, *Methanocella*) were also found in these soils. Mirza et al. (2014) suggested that archaeal biological N fixation could play an important role in the Amazon rainforest.

Biological N fixation rates in tropical forests (15 to 36 kg N ha^-1^ yr^-1^) are similar to or higher than estimates for their temperate counterparts (7 to 27 kg N ha^-1^ yr^-1^), which are subjected to strong N limitation (Cleveland et al., 1999). Houlton et al. (2008) suggest that diazotrophs could be favored in tropical forests because they acquire enough N to maintain higher extracellular phosphatase activity, which is required to overcome P limitation in these ecosystems, and because the temperature in tropical environments is near the optimum for biological N fixation. Furthermore, until recent years it was thought that free-living diazotrophs were the dominant form of N-fixing bacteria in temperate forest soils and symbiotic diazotrophs were the dominant form of N-fixing bacteria in tropical forest soils, where leguminous trees are common (Cleveland et al., 1999). However, recent studies in tropical forests indicate that free-living N-fixing bacteria in litter and soil may be more important than previously thought (Vitousek and Hobbie, 2000; Cusack et al., 2009; Hedin et al., 2009; Reed et al., 2013). Forest canopy communities, especially lichens, mosses, and other epiphytes associated with cyanobacteria, can also provide significant inputs of N to tropical forests (Matzek and Vitousek, 2003; Benner et al., 2007). Cusack et al. (2009) estimated N inputs via biological fixation by free-living microbes from above- and belowground components of two tropical forests in Puerto Rico, and assessed the response to increased N availability using an N fertilization experiment. Their results showed that free-living N fixation in soils was suppressed by N fertilization (50 kg N ha^-1^ yr^-1^) and was significantly positively correlated with soil moisture, similar to findings in a tropical rainforest in Panama (Barron et al., 2008).

Tropical ecosystems are characterized by N fixation hotspots —zones where N fixation rates are much higher than average— that may reflect the very small-scale spatial heterogeneity of abiotic factors affecting the dynamics of the diazotroph community (Reed et al., 2010). While many tropical soils are generally nutrient-poor, there is a great deal of spatial and temporal heterogeneity in nutrient availability even on highly weathered soils (Townsend et al., 2008). The distribution and activity of N-fixing organisms in tropical forest soils are mainly regulated by moisture (Cusack et al., 2009), C/N ratio (Vitousek et al., 2002), and the availability of both P and molybdenum (Mo; Pearson and Vitousek, 2002; Benner et al., 2007; Reed et al., 2007; Barron et al., 2008; Wurzburger et al., 2012). Parent material can also drive the abundance of diazotroph communities in tropical soils. Stone et al. (2015) found that Inceptisol soils (primary-mineral rich soils) may provide a more favorable environment for diazotroph communities (and also denitrifier communities) compared with their Oxisol (acid soils, rich in Fe and Al secondary minerals) counterparts in two tropical forest types in Puerto Rico. Higher N loss rates due to leaching in Inceptisol subsoils could produce a feedback on surface N-cycle processes, increasing microbial N demand and ultimately stimulating N-fixation. In addition, anaerobic conditions can significantly increase rates of biological N fixation because O_2_ inhibits the activity of the nitrogenase enzyme, and thus soil moisture content is an important factor in regulating rates of biological N fixation (Hicks et al., 2003).

Both P and micronutrients play important roles in regulating N fixation in tropical forests. The high P demand of diazotrophs to provide the large amounts of energy required by this process creates the potential for P limitation of N fixation. In addition, P in many lowland tropical forests is bound up with highly weathered soil minerals and organic matter. Microorganisms are able to liberate this P by exuding phosphatase enzymes and their synthesis requires large amounts of N. Thus, N fixation in tropical forests may confer a competitive advantage to diazotrophs by facilitating the uptake of P (Houlton et al., 2008). In line with these assertions, some experimental studies have shown that P addition stimulates this process (Benner et al., 2007; Reed et al., 2007), as well as increases the abundance and diversity of free-living diazotrophs in these ecosystems (Reed et al., 2010). Similarly, Mo is a critical component of the most common form of nitrogenase and some studies conducted in the tropical rainforest of Panama have suggested Mo limitation of N fixation (Barron et al., 2008; Wurzburger et al., 2012). Reed et al. (2013) performed a full-factorial litter incubation experiment to explore P and Mo controls over free-living N fixation rates from a Costa Rican tropical rainforest. Results showed that variation in P concentration (and not Mo) was positively correlated with N fixation rates. These authors asserted that more data are clearly needed to draw firm conclusions about general patterns in P vs Mo limitation of N fixation in these soils.

### Nitrifying Microorganisms

Nitrification is the stepwise aerobic oxidation of 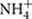 or ammonia (NH_3_) to nitrite (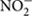) and 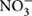. This key process plays an important role in regulating N availability and loss in terrestrial ecosystems, because it can cause 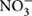 leaching to groundwater and N_2_O production directly (via chemical decomposition of hydroxylamine) and indirectly (via denitrification and nitrifier denitrification). Nitrification in soils is divided into autotrophic nitrification and heterotrophic nitrification. The former is mainly carried out by chemoautotrophic ammonium-oxidizing bacteria (AOB, represented by *Nitrosomonas*, *Nitrosospira*, and *Nitrosococcus* species) and archaea (AOA, represented by *Nitrososphaera* and *Nitrosotalea* linages from the phylum Thaumarchaeota), and nitrite-oxidizing bacteria (NOB, represented by *Nitrobacter*, *Nitrospina*, *Nitrococcus*, and *Nitrospira* species). Heterotrophic nitrification is carried out by certain heterotrophic bacteria and fungi with the potential to oxidize both organic and inorganic N compounds (Hayatsu et al., 2008). Although it is widely accepted that autotrophic nitrification in soils is the prevalent nitrification process, high nitrification rates in tropical and subtropical acidic soils suggest that heterotrophic nitrification may be important in these soils (Pett-Ridge et al., 2013; Zhang et al., 2013), because it is less impacted by low pH than autotrophic nitrification (de Boer and Kowalchuk, 2001). In fact, using the ^15^N tracing technique, both Zhang et al. (2011) and Zhu et al. (2013) found that heterotrophic nitrification was the dominant process producing 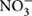 in subtropical acidic coniferous forest, where autotrophic nitrification rate was very low. Using the ^15^N tracing method and a selective biomass inhibitor (fungal inhibitor: cycloheximide and bacterial inhibitor: streptomycin), Zhu et al. (2015) identified that fungal rather than bacterial pathways dominate heterotrophic nitrification in the same forest soils, suggesting that fungi may be the important driver for heterotrophic nitrification in acid forest soils of subtropical China. However, the ecology of heterotrophic nitrifier microorganisms has not yet been investigated in tropical forest soils.

Nitrifier denitrification is an alternative pathway and involves the oxidation of NH_3_ to 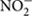 and its subsequent reduction via NO to N_2_O by AOB (Colliver and Stephenson, 2000), particularly by *Nitrosospira* taxa (Shaw et al., 2006). Recent studies have suggested that this process may contribute considerably to N_2_O fluxes from soils with high N content and low pH and oxygen pressure (Wrage et al., 2004; Kool et al., 2010; Gharahi Ghehi et al., 2012). These conditions are typical in tropical forest soils, thus we speculate that nitrifier denitrification could play an important role in these ecosystems. However, to our knowledge, no studies directly related to this process have been conducted in tropical forests soils.

Microbial ammonia oxidation is the first and rate- limiting step of autotrophic nitrification, which may explain why the few studies on microbial nitrification in tropical forest soils have mainly focused on this process. Ammonia-oxidizing microorganisms oxidize NH_3_ to 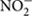 by using the ammonia monooxygenase enzyme, the α-subunit of which is encoded by the *amoA* gene, a common genetic marker for nitrification (Rotthauwe et al., 1997; Treusch et al., 2005). It was presumed that chemolithotrophic Gamma- and Betaproteobacteria were primarily responsible for this process in soils (Kowalchuk and Stephen, 2001). However, recent studies have demonstrated that AOA are ubiquitous constituents of terrestrial environments (Leininger et al., 2006; Pester et al., 2012; Zhalnina et al., 2012; de Gannes et al., 2014) and their discovery has changed the paradigm of aerobic nitrification. The ecology of AOA remains under investigation, and there is not a simple relationship between NH_3_ oxidation rates and the relative abundances of AOA and AOB (Nicol and Schleper, 2006; Taylor et al., 2012).

In soils, drivers of niche differentiation of AOA vs AOB communities have centered largely on pH, NH_3_ concentrations and N organic forms. High abundances of archaeal *amoA* genes have been reported in many acidic soils (Leininger et al., 2006; Boyle-Yarwood et al., 2008; Chen et al., 2008; Stopnišek et al., 2010; Isobe et al., 2012) and multiple studies suggest that some groups of AOA may prefer a pH below 5.5 (Nicol et al., 2008; Gubry-Rangin et al., 2010; Yao et al., 2013). In strongly acidic soils, AOA abundance increases or remains unchanged with decreasing pH, while AOB abundance decreases (He et al., 2007; Yao et al., 2011; Taylor et al., 2012). However, in neutral to alkaline soils, correlations of AOA or AOB abundance to pH have not been consistent (Shen et al., 2008; Jia and Conrad, 2009). Investigations have also indicated a preference of soil AOA communities for low NH_3_ levels (Wessén et al., 2010; Verhamme et al., 2011) and low organic nutrient conditions (Erguder et al., 2009).

Database surveys focused on soils from temperate regions have revealed a strong biogeographical component to the distribution of AOA and AOB phylotypes (Fierer et al., 2009a; Pester et al., 2012). However, in tropical soils very little is known about ammonia-oxidizing microorganisms and how environmental factors affect their activity. Many tropical forest soils tend to be wetter, have smaller annual fluctuations in temperature, lower pH, high available N and more reduced conditions than temperate forest soils, and this may impose different environmental conditions (and require different traits) for ammonia-oxidizing organisms. For instance, *Nitrosospira* cluster 2 has been found in great abundance in acidic soils (Boyle-Yarwood et al., 2008), but it has been suggested that it may be more common in cold-temperate forest soils than in warmer tropical soils (Avrahami and Conrad, 2005; Fierer et al., 2009a). So far, ammonia oxidizer *amoA* sequences from clone libraries have been described from only a few wet tropical forest soils from Trinidad (de Gannes et al., 2014), Puerto Rico (Pett-Ridge et al., 2013), and Costa Rica (Carney et al., 2004), as well as from two different subtropical forest soils in China (Yao et al., 2011; Isobe et al., 2012). Only three of these studies have successfully amplified bacterial *amoA* sequences from these soils, with *Nitrosospira* and *Nitrosomonas* the primary taxa (Carney et al., 2004; Yao et al., 2011; de Gannes et al., 2014). The majority of archaeal *amoA* sequences described in these soils clustered with sequences from *Nitrososphaera* and *Nitrosotalea* (Pester et al., 2012; Pett-Ridge et al., 2013; de Gannes et al., 2014).

Similar to many acidic temperate forest soils, AOA populations have been inferred to be the primary group driving nitrification in tropical forest soils (Yao et al., 2011; Lammel et al., 2015). Furthermore, Isobe et al. (2012) and Pett-Ridge et al. (2013) were unable to amplify bacterial *amoA* genes from acidic tropical forest soils (**Supplementary Table S2**). At least two other studies examining acidic forest soil have found a similar pattern of absence of bacterial *amoA* genes (Stopnišek et al., 2010; Levičnik-Höfferle et al., 2012). Thus, one possible explanation for the dominance of AOA in these soils is the relatively low soil pH (Nicol et al., 2008; Gubry-Rangin et al., 2010; Yao et al., 2013). For example, Yao et al. (2011) observed that the ratio of AOA to AOB *amoA* gene abundance increased with decreasing soil pH in subtropical soils in China. Significant relationship between AOA abundance and nitrification potential was also found, indicating that nitrification was mainly driven by AOA in these acidic soils. However, de Gannes et al. (2014) found that AOA abundance predominated over AOB in tropical soils that spanned pHs of 4 to 8 in Trinidad (**Supplementary Table S2**), and the community abundance and structure of AOA was affected primarily by soil N characteristics, but not by soil pH. According to these authors, the effect of pH on nitrification appeared to mainly reflect impacts on AOA or AOB activity, rather than selection for AOA or AOB phylotypes differing in nitrifying capacity. In addition, Jiang et al. (2015) found higher net nitrification rate and abundance of AOA and AOB for Cambisols than for Ferralsols from the same parent material in a subtropical acidic forest in China (**Supplementary Table S2**). These authors also reported higher 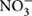-N immobilization for Ferralsols than for Cambisols, which imply that the lower nitrification rate and *amoA* abundance in these Ferralsols may due to high amounts of 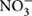-N immobilization stimulated by high Fe oxide concentrations. A potential mechanism of abiotic immobilization of NO_3_ has been postulated, suggesting that Fe plays a key role in 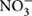-N immobilization by promoting organic N formation from inorganic N (Davidson et al., 2003). Thus, *amoA* genes abundance may be more affected by soil nutrients and Fe oxide contents rather than a low pH in tropical forest soils.

In contrast to temperate forest soils, tropical soils routinely experience fluctuating O_2_ availability over short time scales. This could represent a unique selective habitat for ammonia oxidizers adapted to this O_2_ regime. Laboratory measurements in Puerto Rican forest soils showed elevated 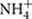 pools and high gross nitrification rates immediately following periods of anaerobiosis, indicating that substantial 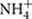 oxidation occurs despite low pH and limited O_2_ availability (Pett-Ridge et al., 2006, 2013). The survival mechanisms of these nitrifiers during periods of prolonged anaerobiosis are not well known. It has been shown that under oxygen-limiting conditions, ammonia oxidizers can use 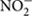 as a terminal electron acceptor (Shrestha et al., 2002), or exploit the oxic-anoxic spatial segregation (Sey et al., 2008). Another explanation is that 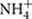 is constantly regenerated via DNRA due to the redox fluctuation conditions in tropical soils (Silver et al., 2001; Templer et al., 2008), and with a subsequent influx of O_2_, this 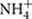 becomes reavailable to ammonia oxidizer communities. On the other hand, AOA populations appear to be insensitive to O_2_ availability (Zhalnina et al., 2012). Tropical soil AOA not only tolerate extended periods of low O_2_ (Pett-Ridge et al., 2013), but can react faster than AOB in alternating aerobic/anaerobic conditions (Chen et al., 2008). It has also been proposed that the pathway of 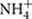 oxidation in AOA may be distinct from the AOB pathway, theoretically requiring only 0.5 O_2_ per 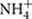 oxidized (Walker et al., 2010). This hypothesized lower O_2_ demand could explain why AOA populations seem to persist more readily under low-oxygen conditions.

A lack of correspondence between nitrification rates and nitrifier community abundance in tropical forest soils has been reported by some investigators (Pett-Ridge et al., 2013; de Gannes et al., 2014), and the reasons for this disconnection are unknown. It is possible that unique ammonia-oxidizers exist in these ecosystems, which can evade detection by the PCR primers developed from currently available sequence data. It is tempting to speculate that the particular conditions of these soils may select for specific lineages of ammonia oxidizers, although much more molecular microbial diversity data from these soils and additional characterization of soil isolates are needed to test this hypothesis.

### Denitrifying Microorganisms

Denitrification is a microbial anaerobic respiration pathway through which 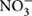 or 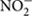 are sequentially reduced to N_2_ via the intermediates NO and N_2_O. It is the major biological process in soils that returns fixed N to the atmosphere and closes the N cycle (Philippot et al., 2007). It is also the primary pathway of NO and N_2_O emissions from soil (Houlton and Bai, 2009). Denitrification consists of four reactions catalyzed by the metallo-enzymes nitrate reductase, nitrite reductase, nitric oxide reductase, and nitrous oxide reductase (Zumft, 1997). Nitrite reductase is the key enzyme of denitrification, since it catalyzes the reduction of 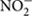 to gas products (Henry et al., 2004), which is the reaction that limits the rate of the denitrification process (Zumft, 1997). Several studies have used genetic markers for denitrification, including *narG* and *napA* (markers for nitrate reductase), *nirK* and *nirS* (markers for nitrite reductase), *norB* (a marker for nitric oxide reductase) and *nosZ* (a marker for nitrous oxide reductase), to study the diversity of denitrifying microorganisms in various environments (Henry et al., 2006; Kandeler et al., 2006; Smith et al., 2007; Yu et al., 2014). Denitrifier organisms can have different combinations of genes involved in the denitrification pathway (Zumft and Kroneck, 2006; Jones et al., 2008). Likewise, while some microorganisms harbor all denitrification enzymes and can potentially perform the complete pathway, others lack the nitrous oxide reductase gene and produce only N_2_O as the denitrification end product (Philippot et al., 2011). Furthermore, recent studies have identified non-denitrifying N_2_O reducers with atypical *nosZ*, which are potential contributors to N_2_O reduction in soils (Sanford et al., 2012; Jones et al., 2013). Thus, understanding the ecology of this functional guild is potentially important for mitigating N_2_O emissions from soils. This newly identified clade of N_2_O reducers is diverse and widespread in terrestrial environments (Jones et al., 2013), but the ecology of this group in tropical forest soils is still unknown.

Denitrification is distributed among a taxonomically diverse group of microorganisms, which have acquired the ability to denitrify via different evolutionary mechanisms (Tiedje et al., 1983; Jones et al., 2008). This process in soil is mainly performed by facultative aerobic heterotrophic bacteria, such as some species of *Pseudomonas*, *Bacillus* and *Paraccocus*, and autotrophic bacteria, such as *Thiobacillus denitrificans* (Philippot et al., 2007; Demanèche et al., 2009). Denitrification is also found among a few archaea (Cabello et al., 2004; Bartossek et al., 2010) and fungi (Shoun et al., 1992; Hayatsu et al., 2008), including Ascomycota (e.g., *Fusarium oxysporum*, *Fusarium solani*, *Cylindrocarpon tonkinense* and *Gibberella fujiuroii*) and Basidiomycota (e.g., *Trichosporon cutaneum*). Fungi generally lack N_2_O reductase (Shoun et al., 1992), suggesting that these organisms are responsible for a large portion of N_2_O emission from soil. Despite the frequent observation that tropical microbial communities in soil are often dominated by fungi (Hawksworth, 2012), their contributions to N_2_O emissions from tropical forest soils have not yet been addressed. The polyphyletic distribution of denitrifying genes can result in their co-occurrence with N-fixation and ammonia-oxidation genes in many microbes (Bedmar et al., 2005; Hayatsu et al., 2008). Many diazotrophic bacteria, such as some species of *Azospirillum* and *Bradyrhizobium*, can denitrify (Rosch et al., 2002). Similarly, AOB belonging to either the genus *Nitrosospira* or *Nitrosomonas* have been shown to be capable of denitrification (Shaw et al., 2006).

Understanding the link between ecosystem scale denitri fication rates and the ecology of the microorganisms that mediate this pathway has been identified as a critical research goal for the mitigation of climate change (Richardson et al., 2009). For this reason, numerous studies have been conducted on the microbially mediated denitrification process in temperate soils, especially its role in N losses and its sensitivity to various environmental factors (Hofstra and Bouwman, 2005; Katsuyama et al., 2008; Attard et al., 2011; Dandie et al., 2011). Despite the growing evidence that denitrification mechanisms and denitrifier communities in tropical forest soils may be very different from those of temperate soils (due to their unique soil characteristics and environmental conditions; Zhang et al., 2009; Xu et al., 2013), few studies exist that document the relationship between denitrifier abundance and total N gas production in tropical and subtropical soils (Liu et al., 2013b; Yu et al., 2014; Zhang et al., 2014; Lammel et al., 2015; **Table 1**; **Supplementary Table S2**). Furthermore, contradictory results have been published about the importance of denitrification as a significant route of N loss in tropical ecosystems. Some studies have shown that tropical forest soils are characterized by generally lower denitrification capacity than temperate forest soils, with greater variability both temporally and spatially due to heterogeneity in edaphic conditions (Robertson and Tiedje, 1988; Xu and Cai, 2007; Zhang et al., 2014). However, tropical soils are considered to be the largest natural sources of NO and N_2_O due to the combination of climatic factors, soil properties, and rapid rates of N cycling (Davidson et al., 2001; Neill et al., 2005; Stehfest and Bouwman, 2006). The conceptual “hole-in-the-pipe” model gives a simplified view of the main controls on N_2_O and NO fluxes (Firestone and Davidson, 1989). In this model, the rate of nitrification and denitrification is conceptualized as a pipe, while the relative ratio of NO and N_2_O to total gas produced is conceptualized as a hole. According to Zhang et al. (2014), N flowing “through the pipe” is significantly less in subtropical forest soils than in temperate forest soils. However, the “holes in the pipe” are much bigger in subtropical forest soils than in temperate forest soils, resulting in a larger amount of NO and N_2_O gases “leaking out of the pipe”. High redox potential (resulting from enrichment of iron and aluminum oxides), low pH and high N availability are the key factors affecting the amount of N flowing through the pipe and the size of the holes in the pipe. These factors may suppress complete denitrification in humid tropical soils and enhance NO and N_2_O ratios during denitrification (Zhang et al., 2009).

The quantification of denitrification genes to elucidate the relative importance of abiotic environmental factors versus denitrifier distribution has been recently achieved in tropical forest ecosystems (Paula et al., 2014; Yu et al., 2014; Zhang et al., 2014; Lammel et al., 2015; Stone et al., 2015; **Table 1**; **Supplementary Table S2**). These studies indicate that many soil properties can affect denitrifier community attributes and denitrification rates in tropical soils, including parent material, pH, redox potential, O_2_ partial pressure, available organic C and N, and 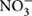 concentration. For example, the abundances of denitrification genes (*nirS* and *nosZ*) were positively correlated with soil C, N, and P concentrations and were more abundant in the Inceptisol compared with the Oxisol soil type in humid tropical forests in Puerto Rico (Stone et al., 2015), suggesting more dynamic N transformation processes in the sandier soil sitting on a younger, more rapidly weathering parent material. Yu et al. (2014) found that the abundance of both *norB* and *nosZ* gene copies were positively correlated with soil pH, and NO and N_2_O fluxes from acidic forest soils (pH 4.4-4.7) were lower than in farmland soils with higher pH (5.0-5.3) in a subtropical region of China. On the other hand, Zhang et al. (2014) showed that redox potential, rather than soil pH or organic C, was the key soil variable influencing denitrification capacity and the abundance of denitrifiers in subtropical forest soils from China. Plant species composition has also been suggested as an important factor in determining the distribution and abundance of denitrifiers in soil (Bremer et al., 2007). So far, only one study has addressed the influence of plant species composition on the abundance of soil N functional genes in tropical soils (Reverchon et al., 2015). These authors found that inter-planting teak with fluegge increased N stocks and the relative abundance of denitrification genes compared to teak mono-plantations in the Salomon Islands, suggesting that mixed species plantations may potentially alleviate N losses and favor N retention.

Lammel et al. (2015) found that pristine rainforest soils in Amazonia showed higher N content and abundances of denitrifier genes (*nirK* and *nosZ*) than pasture and crop fields, which correlated with N_2_O emissions. However, Liu et al. (2013b) found no correlation between denitrifier abundance and N_2_O fluxes in a tropical forest soil in Australia, suggesting that soil C and N substrate availability and soil environmental factors (pH and moisture) rather than denitrification gene abundance control N_2_O fluxes in this ecosystem. Studies in other soil environments have also reported that changes in denitrification rate were not associated with changes in denitrifier community structure (Hallin et al., 2009; Attard et al., 2011). This may be attributed to the high functional redundancy commonly found in phylogentically diverse groups such as denitrifying bacteria. A further understanding of this topic requires research on the expression of denitrification genes (e.g., via RNA-based approaches that target physiologically active populations in denitrification) and its relation to denitrification rates, as well as to separate N_2_O produced through denitrification from nitrifier denitrification.

It is well known that O_2_ (Bollmann and Conrad, 1998), organic C and 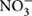 availability (Klemedtsson et al., 2005), low pH (Šimek et al., 2002), or sudden onset of anoxia (Morley et al., 2008) can also influence denitrification rates and negatively affect the reduction of N_2_O to N_2_. However, the mechanisms that increase gene transcription and enhance N_2_O reductase activity have not been researched in detail in tropical forest soils. For example, soil pH is one of the main factors controlling broad-scale microbial activity and the stoichiometry of denitrification (Šimek et al., 2002; Liu et al., 2010). This process in acid soils, such as typical tropical forest soils, seems to be particularly disposed to high N_2_O/N_2_ ratios (Šimek et al., 2002; Zhu et al., 2013), apparently due to impaired functioning of N_2_O reductase at low pH (Liu et al., 2010). However, contradictory results have been published on the relationship between soil pH and denitrification rates in tropical and subtropical acid soils in China. Xu and Cai (2007) showed that denitrification capacity was not correlated with soil pH in humid subtropical soils, but with organic carbon content. Furthermore, no correlation between soil pH and denitrification rate was found in six forest soil types in different climatic zones (temperate, tropical and subtropical forests; Zhang et al., 2009). On the other hand, Yu et al. (2014) reported that low soil pH (<4.7) decreased denitrification rates and NO and N_2_O emissions in subtropical forest soils from China.

Soil moisture is a major driver of N_2_O emissions as it regulates the oxygen availability to soil microbes. N_2_O emissions have their optimum in the range of 70–80% water-filled pore space depending on soil type (Davidson et al., 2000). Since denitrification occurs as soil O_2_ becomes limiting, denitrifiers are likely to be more widespread and resilient in tropical soils due to their temporally and spatially variable redox environment (Pett-Ridge et al., 2006). These taxonomically and physiologically diverse facultative microorganisms preferentially respire O_2_, but can reduce 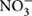 and other partially oxidized N-forms when O_2_ is limiting (Tiedje et al., 1983; Tiedje, 1988). Pett-Ridge et al. (2006) performed a fluctuating redox incubation experiment using tropical forest soils from Puerto Rico and found that denitrification tended to be highest in soils that had been anoxic for several weeks. These results indicated that denitrifiers might dominate in situations where O_2_ is consistently limiting, whereas microbes that mediate DNRA may be more competitive for 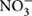 in a fluctuating redox environment (Tiedje, 1988). On the other hand, frequent oxic/anoxic transitions may also inhibit the expression of nitrous oxide reductase, since its activity is more sensitive to O_2_ compared with other denitrifying enzymes (Morley et al., 2008).

## Anthropogenic Factors Influencing Microbial N Cycling in Tropical Forest Soils

Alteration of the N cycle by human activities affects climate, food and energy security, human health, and ecosystem services (Erisman et al., 2013). Anthropogenic factors also have a great potential to alter N processes in tropical forests and significant changes in N dynamics in these forests is expected, including rising 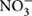 export to ground and surface waters, threatening drinking water quality and increasing eutrophication risks (Matson et al., 1999; Corre et al., 2010). Furthermore, in the coming decades, the tropics will experience unprecedented increases in temperature and significant alterations in precipitation regime, which also have the potential to affect the N cycle (Diffenbaugh and Scherer, 2011; Bai et al., 2013; IPCC, 2013). For example, soil warming may increase microbial metabolism and enzyme activity, resulting in greater organic matter decomposition and gross N mineralization (Koch et al., 2007). Warmer temperatures could also stimulate additional N losses via increased denitrification rates (Bai et al., 2013). However, so far very little is known regarding the response of microbial communities involved in N processes to different components of global environmental change in the tropics. It is beyond the scope of this paper to review the myriad microbial responses to environmental change; rather, we focus our review on the limited currently available research regarding the response of microbial N cycling to anthropogenic factors in tropical forest soils, such as chronic-elevated N deposition, forest perturbation, and land-use change.

### N deposition

Atmospheric deposition of reactive N compounds has increased threefold during the last century due to fossil fuel combustion, artificial fertilizer application and leguminous crops (Vitousek et al., 1997; Gruber and Galloway, 2008). Currently, N deposition adds more than 200 Tg yr^-1^ of N to terrestrial and ocean ecosystems, and is predicted to increase between 50 and 100% by 2030 compared with 2000 (Gruber and Galloway, 2008; Reay et al., 2008). Furthermore, reactive N deposition is dramatically increasing in the tropics because of economic development and increasing biomass burning (Galloway et al., 2004; Hietz et al., 2011), resulting in many unexpectedly negative effects. In fact, elevated N deposition not only occurs in areas with high population densities but also in areas far from the source of N. For example, southern Ecuadorian montane forests receive elevated atmospheric N and P deposition that is largely attributed to biomass burning in the Amazon basin (Fabian et al., 2005). In addition, recent climate models that incorporate N deposition as a part of the CN-coupled models predict a lower net C uptake (37–74%) than values projected by models using C cycle components alone, demonstrating the importance of the N cycle in future climate change (Thornton et al., 2009). Thus, a priority in terrestrial ecosystem research is to determine the fate of N, its interactions with soil microbial communities and its coupling with other biogeochemical cycles, such as the C cycle (Bardgett et al., 2008; Reay et al., 2008; Singh et al., 2010).

The responses of soil N transformations to N addition depend on forest N status, soil characteristics, and climatic factors (Lohse and Matson, 2005; Martinson et al., 2012; Corre et al., 2014). Anthropogenic N deposition has been shown to greatly affect N cycling in temperate and boreal forests, where low availability of N limits primary productivity. These responses have been well studied and summarized in the conceptual model known as the “N saturation hypothesis” (Aber et al., 1998; Galloway et al., 2003). The concept of “N saturation” refers to a state in which N supply exceeds biological demand, resulting in the loss of new N from the ecosystem by biological or physical mechanisms. In this model, atmospheric chronic N deposition in temperate and boreal forests increases soil acidification, stimulates N loss through extensive 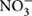 leaching and gaseous N emissions, and produces a switch from N to P limitation and a decline in plant diversity (Aber et al., 1998; Vitousek et al., 1997, 2010). In contrast to temperate forests, tropical forests are generally considered N-rich relative to other nutrients, and thus N deposition is less likely to increase primary productivity of tropical ecosystems, but may alter other aspects of the N cycle (Corre et al., 2010; Hietz et al., 2011). It has been also hypothesized that chronic N deposition will acidify tropical forest soils, further exacerbate P deficiency in highly weathered soils, reduce tree growth and C storage, and negatively affect biodiversity (Matson et al., 1999; Vitousek et al., 2010). Recent evidence suggests that these changes are currently occurring in tropical forests (Hietz et al., 2011; Lu et al., 2014).

Compared with other ecosystem types, tropical forests emitted more N_2_O under N enrichment (on average +739%; Liu and Greaver, 2009), which suggests strong feedbacks of soil N_2_O emissions on increasing atmospheric N deposition in the tropics. However, research on the effect of N deposition in tropical and subtropical forest ecosystems is much more recent, and the temporal scale of such studies much shorter than those in temperate forests. For example, the effect of N deposition on N-saturated tropical forest ecosystems in southern China has been recently studied. Forests in this region have acidified soils, exhibit chronic atmospheric N deposition of 16-38 kg⋅ha^-1^⋅yr^-1^ (Chen and Mulder, 2007; Fang et al., 2011) and high throughfall N input that vary greatly from 11.7 to 65.1 kg N⋅ha^-1^⋅yr^-1^ (Fang et al., 2011). It had been shown that N_2_O emission significantly increases, but soil pH, primary production, litter decomposition and N mineralization significantly decreases in high N addition (100-150 kg N⋅ha^-1^⋅yr^-1^) plots (Lu et al., 2009; Zhu et al., 2013; Wang et al., 2014). These results are consistent with progressive P limitation, which results in higher litter N content and soil N availability. Likewise, a two-year N addition experiment (50-150 kg N⋅ha^-1^⋅yr^-1^) was performed in a humid subtropical bamboo forest in China, which exhibits one of the highest chronic atmospheric N deposition rates in the world (113.8 kg N⋅ha^-1^⋅yr^-1^ in 2009; Tu et al., 2013). In this ecosystem, N deposition accelerated nitrification and denitrification rates, increased hydrolytic enzyme activities, soil N availability and microbial biomass, and decreased soil P availability (Tu et al., 2014). Results suggest that this bamboo forest ecosystem is moving toward P limitation under elevated N deposition, and may increase N_2_O emission and 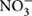 leaching.

The same pattern of increased N-oxide emissions and 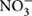 leaching induced by N deposition has been found for montane tropical forests. In Hawaiian montane forests, 11-13 years of high N addition (100 kg N⋅ha^-1^⋅yr^-1^) to an N-limited forest on an Andosol soil and 5-7 years of high N addition (100 kg N⋅ha^-1^⋅yr^-1^) to a P-limited forest on a Ferralsol soil increased gross rates of mineral N production but decreased microbial N retention, leading to higher soil N-oxide emissions and 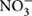 leaching (Hall and Matson, 2003; Lohse and Matson, 2005). Similarly, 1-4 years of high N addition (125 kg N⋅ha^-1^⋅yr^-1^) in an N-limited Panamanian montane forest on an Andosol soil increased gross rates of soil N mineralization and nitrification, leading to increases in soil N-oxide emissions and 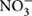 leaching (Corre et al., 2010, 2014). Corre et al. (2014) also showed that this wet montane forest with only 4 years of N addition had comparable N-oxide emissions to an N-rich moist lowland forest in Panama with more than a decade of N addition.

Elevated N deposition in tropical forests may also significantly change soil microbial community composition and activity (Liu et al., 2013a; Tu et al., 2014). There are two primary hypotheses that seek to explain the influence of N addition on microbial community dynamics. The N-mining hypothesis proposes that under N enrichment, microbes reduce decomposition of recalcitrant C in response to their lowered N requirements, resulting in a shift toward labile C decomposition and reduced overall microbial activity (Chen et al., 2014). The copiotrophic hypothesis suggests that N additions decreases the relative abundance of oligotrophic taxa (k-strategists, i.e., slow growing microbes that have high substrate affinity and can catabolize more recalcitrant C pools), which would be out-competed by more copiotrophic taxa (r-strategists, i.e., fast growing microbes that have high nutritional requirements and preferentially consume labile soil organic C pools; Fierer et al., 2007, 2012a; Ramirez et al., 2012).

Knowledge about the effects of N deposition on particular microbially mediated N processes in tropical forests is still limited and controversial (**Table 1**). Cusack et al. (2009) reported that N fertilization (50 kg N ha^-1^ yr^-1^, which is approximately twice the average projected rate for Central America for the year 2050; Galloway et al., 2004) suppressed soil biological N fixation in two tropical forests in Puerto Rico, similar to findings in a rain tropical forest in Panama (Barron et al., 2008). Isobe et al. (2012) investigated nitrifying communities of acidified subtropical forests soils in southern China and found that archaea rather than bacteria control 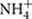 oxidation after long-term N deposition (32-34 kg N⋅ha^-1^⋅yr^-1^). However, it has been reported that rates of heterotrophic nitrification were significantly higher than those of autotrophic nitrification in these forest soils (Zhang et al., 2013). Cong et al. (2015) found that the high relative abundance of both *amoA* and denitrification genes, especially *narG*, in a tropical rainforest soil in China was due to the large amount of soil available N (from 141.4 to 224.5 mg kg^-1^), caused by the high rate of N deposition (over 25 kg N⋅ha^-1^⋅yr^-1^) that affects this ecosystem. In sum, there is evidence indicating that N deposition in tropical forest soils may inhibit biological N fixation (Barron et al., 2008; Cusack et al., 2009), alter N mineralization and nitrification (Zhao et al., 2007; Corre et al., 2010), and induce more N_2_O emissions and 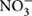 leaching, particularly during the wet season (Hall and Matson, 1999; Corre et al., 2014; Wang et al., 2014). However, the consequences of these changes at the ecosystem scale are not well understood.

**FIGURE 2:**
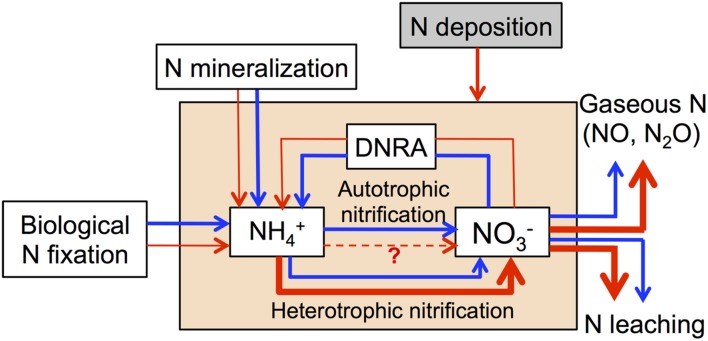
**Conceptual model of how chronic-elevated N deposition affects microbially mediated N processes in N-rich tropical forests soils.** Blue arrows represent N processes without N deposition and red arrows represent N processes with N deposition.

We summarize all available evidence in a conceptual model of how increasing N deposition may affect microbially mediated N processes in N-rich tropical forests soils (**Figure 2**). We hypothesize that chronic-elevated N deposition may lead to a decrease of biological N fixation, N mineralization and DNRA, as well as an increase of heterotrophic nitrification and denitrification, which will lead to high N losses via N leaching and gaseous N production. Furthermore, we also emphasize that these processes are affected by multiple factors such as the initial soil N status, soil type, soil moisture and temperature, elevation (montane or lowland), and landscape position (Martinson et al., 2012; Zhu et al., 2013; Corre et al., 2014; Stone et al., 2015), which may alter the responses of these processes to N deposition.

### Land-Used Change and Forest Perturbation

Land use change is considered the most important factor driving biodiversity losses in tropical areas (Sala et al., 2000). In recent decades, tropical forests have undergone rapid conversion to agriculture, with 80 million hectares of forest cleared in the 1980s and 1990s (Gibbs et al., 2010). Logging and forest conversion result in a series of ecosystem alterations that include an increase in belowground productivity (Cenciani et al., 2009), changes in soil chemistry (McGrath et al., 2001; Murty et al., 2002), and a more open N cycle (Davidson et al., 2007). Following deforestation, not only plant biomass but also the soil stocks of total C and N can become mobilized and be emitted in the form of greenhouse gases (Neill et al., 2005; Galford et al., 2010; Arai et al., 2014). Among these emissions, N_2_O has a global warming potential for 100 years 265-298 times greater than that of the equivalent amount of CO_2_ (IPCC, 2013) and promotes ozone destruction in the stratosphere. Thus, understanding the controlling factors of N_2_O emission is crucial for quantifying the effects of land-use change on the N cycle and for managing and mitigating the environmental consequences associated with N pollution (Galloway et al., 2004).

It has been shown that land use change in the tropics also alters soil bacterial (da Jesus et al., 2009; Rodrigues et al., 2013; Ranjan et al., 2015), fungal (Mueller et al., 2014), and archaeal (Taketani and Tsai, 2010) community composition. For example, a recent study showed that the conversion of Amazonian forest to pasture impacts microorganisms, resulting in biotic homogenization of communities (Rodrigues et al., 2013). Various studies have suggested that deforestation of tropical forests for agriculture and agroforestry decreases microbial biomass and alters the soil microbial composition (Bossio et al., 2005; Arai et al., 2014; Krashevska et al., 2015). However, the impact of land use changes on soil N processes and the microorganisms responsible for these processes has been limited to only a few studies (e.g., Carney et al., 2004; Yu et al., 2014; Lammel et al., 2015; **Table 1**; **Supplementary Table S2**).

So far, only one study has integrated measures of greenhouse gas emissions and the abundance of multiple functional and phylogenetic genetic markers (*nifH, amoA, nirK, nirS, norB, nosZ, mcrA, pmoA*, and *16S/18S rRNA*) with environmental parameters to predict the response of biogeochemical processes to land use change in Amazonian soils (Lammel et al., 2015). Results showed that land use change from forest to agriculture reduced the abundance of different functional microbial groups related to C and N cycles in the wet season, but its effects were less significant after the dry winter fallow. Paula et al. (2014) studied the influence of land use change from forest to pasture on functional gene diversity, composition and abundance in Amazon rainforest soil microbial communities. They found that genes linked to C and N cycling were the most altered by deforestation. Carney et al. (2004) also observed that soil AOB diversity and composition changed significantly with land use in the tropical lowland of Costa Rica, and these changes were significantly associated with shifts in the potential rates of nitrification. The work of Mirza et al. (2014) is one of the first studies using the marker gene *nifH* to assess the response of free-living diazotrophs to deforestation in the Amazon rainforest. They reported a shift in the diazotrophic community composition and an increase in *nifH* gene abundance following forest-to-pasture conversion. They suggested that the response of diazotrophs to land use change is a consequence of changes in plant communities, particularly the higher N demand of pasture for supporting aboveground plant growth. Yu et al. (2014) also found that the abundance of denitrifying genes (*narG, nirK, norB*, and *nosZ*) was significantly affected by land-use change in subtropical soils of China.

## Conclusion and Perspectives

Most studies of the consequences of N cycle alteration induced by anthropogenic environmental changes have been performed in temperate forests where biological processes are limited by N supply. However, the response of N processes to environmental change in tropical forest soils is largely unknown. In order to better understand, predict and mitigate global environmental changes, there is an urgent need to clearly understand the microbial control of greenhouse gas emissions and its interactions with environmental factors. Furthermore, a deeper understanding of microbially mediated N processes in tropical forest soils is crucial as N cycling in these soils impacts food production for a large part of the world, as well as the dynamics of global climate change.

This review reveals large knowledge gaps regarding micro bially mediated N processes in tropical forest soils, gaps that represent critical directions for future research. We propose several topics of research that should be prioritized (**Box 1**). For example, to more accurately predict the response of N cycling to environmental change in tropical forest ecosystems, additional data regarding how N cycling is regulated is needed from a much broader range of tropical forests than currently studied, as well as improvements in our ability to quantify *in situ* the rates of the primary N processes in these soils. Likewise, it is important to carry out studies in tropical forest soils of recently discovered processes, such as anammox and codenitrification. In addition, research is required to link these processes with soil microbial community characteristics and the functional roles of microbial extracellular enzymes, in order to provide mechanistic insights into microbial regulation of the N cycle in tropical forest ecosystems.

**Box 1. Future research on microbial ecology of the N cycle in tropical forest soils.**

• The contributions of different microbial subpopulations to the N cycle.
• The ecology of recently discovered processes, such as anammox and codenitrification.
• Modeling microbial regulation of the N cycle.
• Network analyses to explore links between environmental factors and microbial control of the N cycle.
• Links between specific microbial metabolic processes and the C and N cycles.
• The interactive effects of C, N, and P on the soil microbial community.
• The influence of precipitation and soil warming on the community structure of N-cycling microorganisms.
• The influence of soil spatial heterogeneity on microbial community functions and N cycling regulation.
• The impact of disturbance on microbial community structure and function and its consequences for the N processes at global scales.
• Microbial community responses to multiple experimental climate change drivers.
• Mechanisms regulating the activity and interaction between primary producers and microorganisms controlling N cycling (plant-microbial feedbacks for the regulation of N).

## Author Contributions

SP and BB designed and conceived the study. SP produced the first draft of the manuscript, and both authors edited the manuscript.

## Conflict of Interest Statement

The authors declare that the research was conducted in the absence of any commercial or financial relationships that could be construed as a potential conflict of interest.
