# Supplementary material for: Ecology of Nitrogen Fixing, Nitrifying, and Denitrifying Microorganisms in Tropical Forest Soils
Source: Front Microbiol. 2016 Jul 5;7:1045. doi: 10.3389/fmicb.2016.01045 (PMC4932190; doi:10.3389/fmicb.2016.01045)
Supplement: Supplementary file 1 [file Table_1.DOCX]

**SUPLEMENTARY TABLES**

Table S1. Main differences between tropical and temperate forests.

| **Main characteristics** | **Tropical forests** | **Temperate forests** |
| --- | --- | --- |
| Climatic conditions | Higher rainfall (1,000-5,000 yearly) and temperature (> 20 ˚C), and lower seasonality | Lower rainfall (500-2,000 yearly) and greater seasonality, with temperatures ranging from -15 ˚C to 30 ˚C |
| Biodiversity | The greatest diversity of organisms | Less diverse |
| Productivity and decomposition | Higher (eg: average NPP of 1,600-2,200 g m^-2^ yr^-1^) | Lower (eg.: average NPP of 1,200-1,300 g m^-2^ yr^-1^) |
| Dominant plants | High diversity, abundance and heterogeneity of legumes | Dominance of conifers and deciduous trees |
| Soil type | Ultisols, Oxisols | Alfisols, Spodosols |
| Soil conditions | Low pH (3.5-6), lower organic matter accumulation, P-limited*, large amounts of Fe oxides and plant litter material | Greater range of pH, higher organic matter content |
| N cycle | N-rich system* with open dynamic N cycle and greater rates of N cycling | N-limited system with internal N cycling |
| Soil N transformations | High N fixation rates and N loss by nitrate leaching and N gaseous fluxes | Low potential for N fixation and N losses (mainly in form of DON) |
| N_2_O/N_2_ | Higher (big source of N_2_O: 3 Tg N yr^-1^) | Lower (N_2_O emission of 0.05-1 Tg N yr^-1^) |
| Microbial activity | High activity yearly | Varies seasonally |
| N fixing microbes | Great abundance of symbiotic and free-living diazotrophs. Activity mainly regulated by soil moisture and both P and Mo availability | Dominance of free-living diazotrophs. Gene abundance links to soil organic C |
| Nitrifying microbes | Soil N availability and fluctuating O_2_ promote higher abundance of AOA | Soil pH and NH_3_ availability create niche separation between AOA and AOB |
| Denitrifying microbes | Not clear relationship between genes abundance and soil properties | Genes abundance links to soil pH, moisture, organic C and various N species |

* Not for montane tropical forests

Table S2. Abundance of N functional genes in tropical forest soils (for more information, see Table 1).

| Process | Encoding enzyme | Gene | Primers* | Gene abundance (copies . g^-1^ soil) | References** |
| --- | --- | --- | --- | --- | --- |
| N fixation | Nitrogenase | *nifH* | F/Rb^1^  PolF/PolR^2^ | 2.1 x 10^7^ (Amazonian PTF)  2.1 x 10^4^ (Amazonian PTF)  5.0 x 10^4^ (Amazonian STF) | Lammel et al. (2015)  Mirza et al. (2014) |
| Ammonia oxidation | Ammonia monooxygenase  Hydroxylamine oxidoreductase | *AmoA*- Archaea  *AmoA*-Bacteria  *hao* | F/R^3^  F/R^3^  AF/AR^4^  F/R^3^  CrenamoA23f/A616r^5^  CrenamoA23f/A616r^5^  1F/2R^6^  1F/2R^6^  BF/BR^4^  1F/2R^6^  1F/2R^6^  1F/2R^6^ | 5.8 x 10^5^ (Amazonian PTF)  1.9 x 10^7^ (subTF, Ferrasol, China)  6.3 x 10^7^ (subTF, Cambisol, China)  1.2 x 10^4^ (seasonal TF, Trinidad)  5.9 x 10^5^ (montane rain TF, Puerto Rico)  5.2 x 10^5^ (montane wet TF, Puerto Rico)  1.1 x 10^5^ (rain subTF, Puerto Rico)  1.2 x 10^9^ (old subTF, China)  9.3 x 10^8^ (secondary pine subTF, China)  3.2 x 10^8^ (mixed subTF, China)  5.5 x 10^4^ – 1.2 x 10^6^ (secondary pine subTFs, China)  4.8 x 10^5^ (Amazonian PTF)  4.5 x 10^6^ (subTF, Ferrasol, China)  7.8 x 10^6^ (subTF, Cambisol, China)  Low abundance and infrequently detected (seasonal TF, Trinidad)  BDL (TFs, Puerto Rico)  BDL (subTF, China)  BDL - 2.6 x 10^4^ (secondary pine subTFs, China)  No available data | Lammel et al. (2015)  Jiang et al. (2015)  De Gannes et al. (2014)  Pett-Ridge et al. (2013)  Isobe et al. (2012)  Yao et al. (2011)  Lammel et al. (2015)  Jiang et al. (2015)  De Gannes et al. (2014)  Pett-Ridge et al. (2013)  Isobe et al. (2012)  Yao et al. (2011)  No references for TF |
| Nitrite oxidation | Nitrite oxidoreductase | *nxr* |  | No available data | No references for TF |
| Nitrate reduction | Periplasmic dissimilatory nitrate reductase  Membrane-bound dissimilatory nitrate reductase | *napA*  *narG* | 1960F/2650R^7^  1960F/2650R^7^  G-R/G-F^8^ | No available data  6.0 x 10^5^ – 3.0 x 10^6^ (secondary coniferous subTF, China)  3.7 x 10^7^ (subTF, China)  3.5 x 10^7^ (wet sclerophyll forest, Australia) | No references for TF  Yu et al. (2014)  Zhang et al. (2014)  Liu et al. (2013b) |
| Nitrite reduction | Cytochrome cd1 nitrite reductase  Copper nitrite reductase | *nirS*  *nirK* | Cd3aF/R3cd^9^  4QF/6QR^10^  F1aCu/R3Cu^9,11^  F1aCu/R3Cu^9,11^  F1aCu/R3Cu^9,11^  876/1040^12^ | 2.2 x 10^6^ (Amazonian PTF)  1.6 x 10^6^ (wet sclerophyll forest, Australia)  7.4 x 10^6^ (Amazonian PTF)  1.0 x 10^6^ - 3.0 x 10^6^ (secondary coniferous subTF, China)  4.0 x 10^7^ (subTF, China)  6.3 x 10^7^ (wet sclerophyll forest, Australia) | Lammel et al. (2015)  Liu et al. (2013)  Lammel et al. (2015)  Yu et al. (2014)  Zhang et al. (2014)  Liu et al. (2013b) |
| Nitric oxide reduction | Nitric oxide reductase | *cnorB* | *cnor*B F/R^13^  *cnor*B_B_ F/R^13^  *cnor*B_B_ F/R^13^ | 2.0 x 10^7^ (Amazonian PTF)  8.0 x 10^5^ - 4.0 x 10^6^ (secondary coniferous subTF, China)  1.6 x 10^6^ (subTF, China) | Lammel et al. (2015)  Yu et al. (2014)  Zhang et al. (2014) |
| Nitrous oxide reduction | Nitrous oxide reductase | *nosZ* | 2F/2R^14^  2F/2R^14^  2F/2R^14^  2F/2R^14^ | 2.3 x 10^7^ (Amazonian PTF)  7.0 x 10^6^ - 8.0 x 10^6^ (secondary coniferous subTF, China)  6.0 x 10^7^ (subTF, China)  3.1 x 10^7^ (wet sclerophyll forest, Australia) | Lammel et al. (2015)  Yu et al. (2014)  Zhang et al. (2014)  Liu et al. (2013b) |

*References for primers: ^1^Rosch et al. (2005); ^2^Poly et al. (2001); ^3^Francis, et al., (2005) and Leininger et al. (2006); ^4^de Gannes et al. (2012); ^5^Nicol et al. (2008); ^6^Rotthauwe et al. (1997); ^7^Philippot et al. (2002); ^8^Bru et al (2007); ^9^Throbäck et al. (2004); ^10^Kandeler et al. (2006); ^11^Hallin and Lindgren (1999); ^12^Henry et al. (2004); ^13^Dandie et al. (2007); ^14^Henry et al. (2006).

Abbreviations: TF: Tropical forest; subTF: Subtropical forest; PTF: Primary tropical forest; STF: Secondary tropical forest; BDL: Below detection limit.

**REFERENCES LIST FOR PRIMERS:**

**References from the Table S2 are in the article reference list

Bru, D., Sarr, A., and Philippot, L. (2007). Relative abundances of proteobacterial membrane-bound and periplasmic nitrate reductases in selected environments. *Appl. Environ. Microbiol.* 73, 5971–5974. doi:10.1128/AEM.00643-07.

Dandie, C. E., Burton, D. L., Zebarth, B. J., Trevors, J. T., and Goyer, C. (2007). Analysis of denitrification genes and comparison of nosZ, cnorB and 16S rDNA from culturable denitrifying bacteria in potato cropping systems. *Syst. Appl. Microbiol.* 30, 128–138. doi:10.1016/j.syapm.2006.05.002.

Francis, C. A., Roberts, K. J., Beman, J. M., Santoro, A. E., and Oakley, B. B. (2005). Ubiquity and diversity of ammonia-oxidizing archaea in water columns and sediments of the ocean. *Proc. Natl. Acad. Sci. U. S. A.* 102, 14683–8. doi:10.1073/pnas.0506625102.

de Gannes, V., Eudoxie, G., Dyer, D. H., and Hickey, W. J. (2012). Diversity and abundance of ammonia oxidizing archaea in tropical compost systems. *Front. Microbiol.* 3. doi:10.3389/fmicb.2012.00244.

Hallin, S., and Lindgren, P. (1999). PCR detection of genes encoding nitrite reductase in denitrifying bacteria. *Appl. Environ. Microbiol.* 65, 1652–1657.

Henry, S., Baudoin, E., López-Gutiérrez, J. C., Martin-Laurent, F., Brauman, A., and Philippot, L. (2004). Quantification of denitrifying bacteria in soils by nirK gene targeted real-time PCR. *J. Microbiol. Methods* 59, 327–35. doi:10.1016/j.mimet.2004.07.002.

Henry, S., Bru, D., Stres, B., Hallet, S., and Philippot, L. (2006). Quantitative detection of the nosZ gene, encoding nitrous oxide reductase, and comparison of the abundances of 16S rRNA, narG, nirK, and nosZ genes in soils. *Appl. Environ. Microbiol.* 72, 5181–9. doi:10.1128/AEM.00231-06.

Kandeler, E., Deiglmayr, K., Tscherko, D., Bru, D., and Philippot, L. (2006). Abundance of narG, nirS, nirK, and nosZ genes of denitrifying bacteria during primary successions of a glacier foreland. *Appl. Environ. Microbiol.* 72, 5957–62. doi:10.1128/AEM.00439-06.

Leininger, S., Urich, T., Schloter, M., Schwark, L., Qi, J., Nicol, G. W., et al. (2006). Archaea predominate among ammonia-oxidizing prokaryotes in soils. *Nature* 442, 806–809. doi:10.1038/nature04983.

Nicol, G. W., Leininger, S., Schleper, C., and Prosser, J. I. (2008). The influence of soil pH on the diversity, abundance and transcriptional activity of ammonia oxidizing archaea and bacteria. *Environ. Microbiol.* 10, 2966–2978. doi:10.1111/j.1462-2920.2008.01701.x.

Philippot, L., Piutti, S., Martin-Laurent, F., Hallet, S., and Germon, J. C. (2002). Molecular analysis of the nitrate-reducing community from unplanted and maize-planted soils. *Appl. Environ. Microbiol.* 68, 6121–6128. doi:10.1128/AEM.68.12.6121-6128.2002.

Poly, F., Monrozier, L. J., and Bally, R. (2001). Improvement in the RFLP procedure for studying the diversity of nifH genes in communities of nitrogen fixers in soil. *Res. Microbiol.* 152, 95–103. doi:10.1016/S0923-2508(00)01172-4.

Rosch, C., Mergel, A., and Bothe, H. (2002). Biodiversity of Denitrifying and Dinitrogen-Fixing Bacteria in an Acid Forest Soil. *Appl. Environ. Microbiol.* 68, 3818–3829. doi:10.1128/AEM.68.8.3818.

Rotthauwe, J. H., Witzel, K. P., and Liesack, W. (1997). The ammonia monooxygenase structural gene amoA as a functional marker: molecular fine-scale analysis of natural ammonia-oxidizing populations. *Appl. Environ. Microbiol.* 63, 4704–12. Available at: http://www.pubmedcentral.nih.gov/articlerender.fcgi?artid=168793&tool=pmcentrez&rendertype=abstract.

Throbäck, I. N., Enwall, K., Jarvis, A., and Hallin, S. (2004). Reassessing PCR primers targeting nirS, nirK and nosZ genes for community surveys of denitrifying bacteria with DGGE. *FEMS Microbiol. Ecol.* 49, 401–17. doi:10.1016/j.femsec.2004.04.011.
